# Protocatechuic Acid Ameliorates Cisplatin-Induced Inflammation and Apoptosis in Mouse Proximal Tubular Cells

**DOI:** 10.3390/ijms26094115

**Published:** 2025-04-26

**Authors:** Karim M. Saad, Khaled Elmasry, Babak Baban, Man J. Livingston, Zheng Dong, Marwa E. Abdelmageed, Rania R. Abdelaziz, Ghada M. Suddek, Ahmed A. Elmarakby

**Affiliations:** 1 Department of Oral Biology and Diagnostic Sciences, The Dental College of Georgia, Augusta University, 1450 Laney Walker Blvd, Augusta, GA 30912, USA; kareem_saad4@mans.edu.eg (K.M.S.); khelmasry@augusta.edu (K.E.); bbaban@augusta.edu (B.B.); 2 Department of Pharmacology and Toxicology, Faculty of Pharmacy, Mansoura University, Mansoura 35516, Egypt; marwaelsayed90@mans.edu.eg (M.E.A.); rania200582@mans.edu.eg (R.R.A.); ghmsuddek@mans.edu.eg (G.M.S.); 3 Department of Cellular Biology and Anatomy, The Medical College of Georgia, Augusta University, Augusta, GA 30912, USA; malivingston@augusta.edu (M.J.L.); zdong@augusta.edu (Z.D.); 4 Department of Human Anatomy and Embryology, Faculty of Medicine, Mansoura University, Mansoura 35516, Egypt; 5 Charlie Norwood VA Medical Center, Augusta, GA 30904, USA

**Keywords:** BUMPT, protocatechuic acid, cisplatin, inflammation, barrier function

## Abstract

Cisplatin is a highly cytotoxic drug used for the treatment of head, neck, and soft tissue cancers; however, it has nephrotoxic effects that can lead to acute kidney injury. Protocatechuic acid (PCA) is a natural widely available antioxidant found in many fruits such as kiwi, mango, and berries. We have recently shown that PCA reduced renal injury in a mouse model of unilateral ureteral obstruction. The current study aims to investigate the protective effects of PCA in Cisplatin-induced inflammation in vitro in Boston University Mouse Proximal Tubular (BUMPT) cells. BUMPT cells were cultured in complete DMEM. Confluent BUMPT cells were then treated with 20 μM Cisplatin ± PCA 50 or 100 μM for 24 h. PCA treatment showed a dose-depending increase in % cell viability in Cisplatin-treated BUMPT cells. PCA treatment also dose-dependently decreased Cisplatin-induced increases in oxidative stress (ROS and TBARS), inflammation (p-NF-κB and IL-6), and apoptosis (cleaved caspase-3 and % of TUNEL^+^ cells) compared to Cisplatin-only treatment. The reduction in oxidative stress, inflammation, and apoptosis with PCA treatment in Cisplatin-treated BUMPT cells was associated with decreases in tubular physical barrier resistance and the expression of the tight junction protein zonula occludens-1 (ZO-1) when compared to BUMPT cells treated with Cisplatin alone. The current findings suggest that PCA treatment improves tubular barrier function in Cisplatin-treated BUMPT cells via reductions in oxidative stress, inflammation, and apoptosis.

## 1. Introduction

Cisplatin is a platinum-based chemotherapeutic agent effective against a range of tumors, including ovarian, testicular, head, and neck cancers, as well as sarcomas and bone malignancies [1]. Despite its effectiveness, its clinical use is limited by toxic effects on healthy organs, particularly the kidneys, liver, and reproductive system [2,3,4,5,6,7].

Cisplatin carries a significant risk of nephrotoxicity, affecting 20–30% of patients and often leading to acute kidney injury (AKI) [8]. In addition to its direct cytotoxicity, Cisplatin triggers inflammatory responses that exacerbate kidney damage [9,10]. The mechanisms underlying Cisplatin-induced nephrotoxicity are multifactorial and involve oxidative stress, mitochondrial dysfunction, and the activation of inflammatory pathways such as NF-κB, which leads to elevated pro-inflammatory cytokines including IL-6 [11]. These effects are primarily due to the drug’s prolonged retention and accumulation in the kidneys [12], leading to reduced renal blood flow, glomerular filtration, ischemia, and proximal tubular necrosis [9,13]. Thus, understanding the molecular basis of these effects is essential to prevent AKI in patients undergoing Cisplatin therapy.

Protocatechuic acid (PCA) is a natural polyhydroxy compound that is widely distributed in foods such as mustard [14], kiwi fruit [15], blackberries, strawberries [16], and mango [17]. Furthermore, PCA has multiple beneficial pharmacological activities such as antioxidant, anti-inflammatory, and anti-cancer activities. It is widely available and relatively affordable [18,19]. We have recently demonstrated that PCA reduced renal inflammation and injury in murine models of unilateral ureteral obstruction [20].

Given the central role of proximal tubular injury in Cisplatin-induced nephrotoxicity, the current study aims to investigate the protective effects of PCA in Boston University Mouse Proximal Tubular (BUMPT) cells. Specifically, we assessed PCA’s impact on Cisplatin-induced oxidative stress, inflammation, and apoptosis. In addition, we evaluated barrier integrity using ECIS and the expression of tight junction protein ZO-1.

## 2. Results

### 2.1. Effect of PCA Treatment on % Cell Viability in Cisplatin-Treated BUMPT Cells

**Figure 1:**
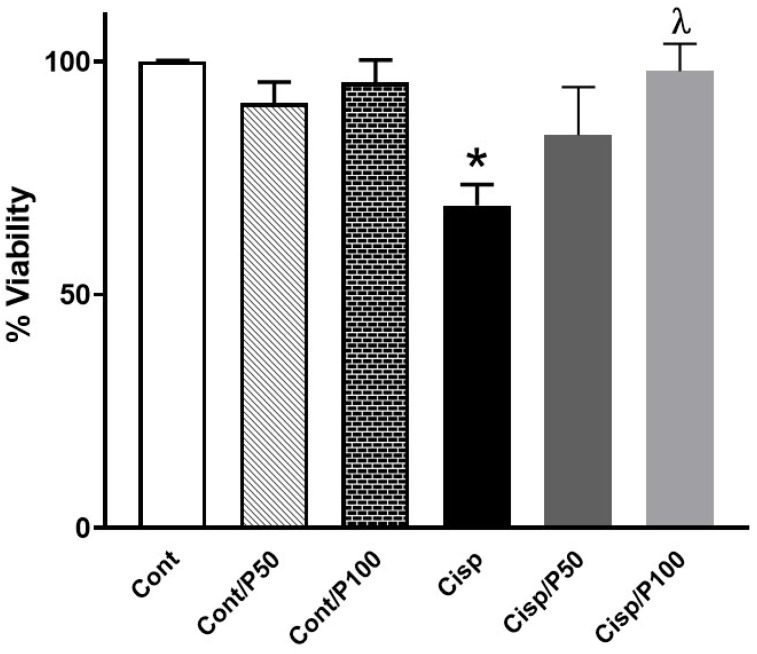
Effect of PCA treatment on % cell viability in Cisplatin-induced injury in BUMPT cells. Data represent means ± SEM (*n* = 7–8 per group). * indicates *p* < 0.05 vs. Cont, Cont/P50, or Cont/P100 and ^λ^ indicates *p* < 0.05 vs. Cisp.

An MTT assay was performed to evaluate % cell viability. The treatment of BUMPT cells with 20 μM Cisplatin for 24 h significantly decreased cell viability compared to the control group (Figure 1). Only 100 μM PCA treatment for 24 h significantly increased cell viability in Cisplatin-treated BUMPT cells, whereas 25 or 50 μM PCA treatment did not significantly increase cell viability in Cisplatin-treated BUMPT cells compared to the Cisplatin-only group (Figure 1). Treating control BUMPT cells with PCA did not significantly affect cell viability at any used concentration compared to the control group (Figure 1).

### 2.2. Effect of PCA on Cisplatin-Induced Changes in Tight Junction Protein (ZO-1) and ECIS

**Figure 2:**
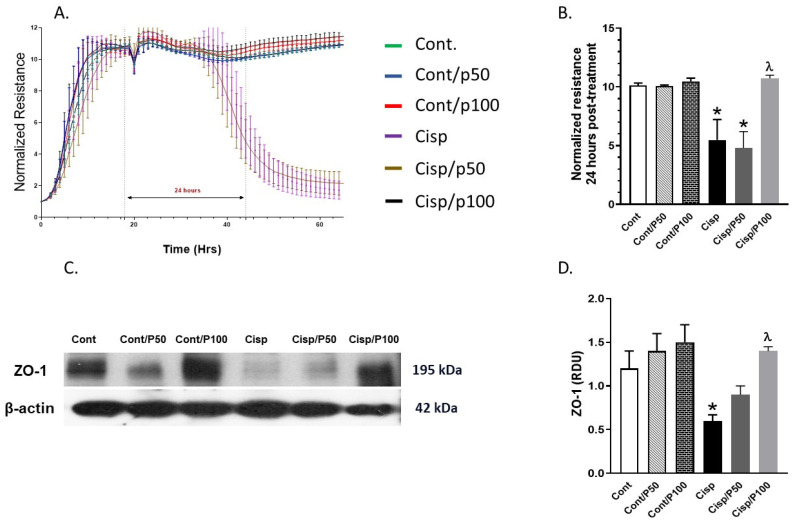
Effect of PCA on Cisplatin-induced changes in normalized resistance (**A**,**B**) and ZO-1 expression (**C**,**D**). Data are means ± S.E.M (*n* = 7–8 per group) * indicates *p* < 0.05 vs. Cont, Cont/P50, or Cont/P100 and ^λ^ indicates *p* < 0.05 vs. Cisp.

PCA treatment alone did not affect normalized resistance or ZO-1 expression in control BUMPT cells (Figure 2A–D), confirming its lack of intrinsic toxicity on tight junction integrity. However, Cisplatin treatment for 24 h significantly reduced normalized resistance (Figure 2A,B) and ZO-1 expression (Figure 2C,D) compared to control cells. Co-treatment with 100 μM PCA significantly restored both normalized resistance (Figure 2A,B) and ZO-1 expression (Figure 2C,D) in Cisplatin-treated cells. In contrast, 50 μM PCA did not significantly reverse the Cisplatin-induced reductions in either parameter.

### 2.3. The Effect of PCA on Cisplatin-Induced Changes in ROS Production and TBARS Concentration in BUMPT Cells

**Figure 3:**
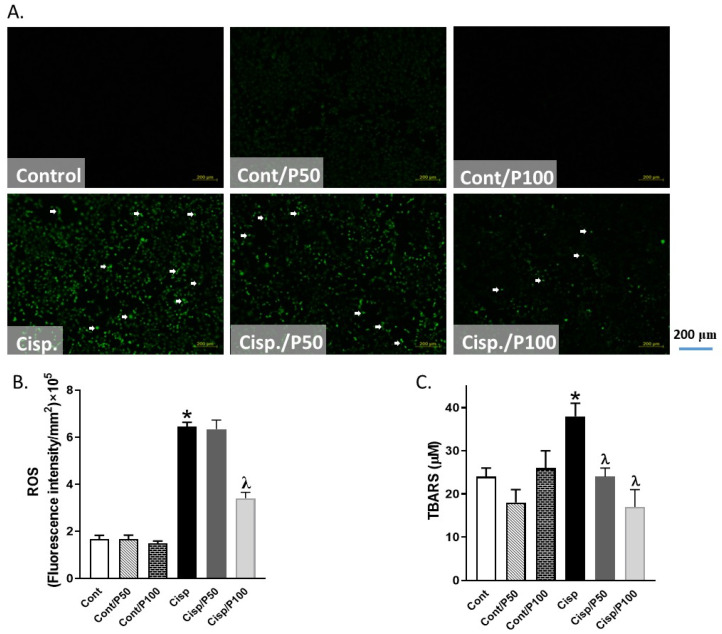
Effect of PCA on Cisplatin-induced changes in ROS production (**A**,**B**) and TBARS levels (**C**) in BUMPT cells. Data represent means ± SEM (*n* = 7–8 per group). White arrows indicate areas of increased green fluorescence corresponding to elevated ROS levels. * indicates *p* < 0.05 vs. Cont, Cont/P50, or Cont/P100 and ^λ^ indicates *p* < 0.05 vs. Cisp.

Treating BUMPT cells with Cisplatin significantly increased ROS production and TBARS levels compared to the control group (Figure 3A–C). Only 100 μM PCA significantly reduced ROS and TBARS in Cisplatin-treated BUMPT cells when compared to Cisplatin-treated BUMPT cells alone. Although 50 μM PCA significantly decreased TBARS levels in Cisplatin-treated BUMPT cells, similar concentrations of PCA failed to significantly reduce ROS production in Cisplatin-treated BUMPT cells (Figure 3A–C).

### 2.4. Effect of PCA on Cisplatin-Induced Changes in IL-6 and p-NF-κB in BUMPT Cells

**Figure 4:**
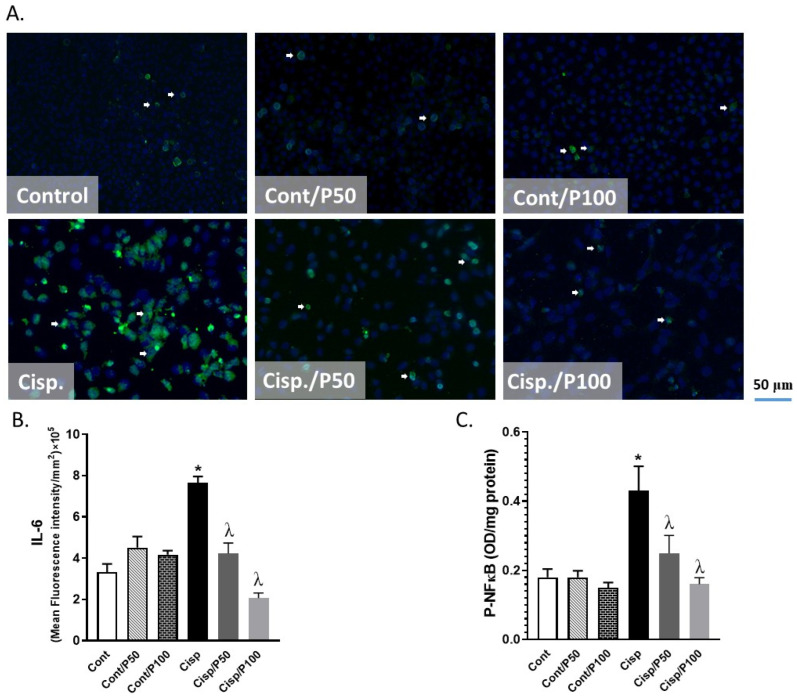
Effect of PCA on Cisplatin-induced changes in expression of IL-6 (**A**,**B**) and p-NF- κB (**C**) in BUMPT cells. Data represent means ± SEM (*n* = 7–8 per group). White arrows indicate areas of green fluorescence corresponding to IL-6 expression with nuclear counterstaining in blue (DAPI). * indicates *p* < 0.05 vs. Cont, Cont/P50, or Cont/P100 and ^λ^ indicates *p* < 0.05 vs. Cisp.

Treating BUMPT cells with Cisplatin significantly increased the levels of IL-6 and p-NF-κB compared to the control group (Figure 4A–C). The PCA treatment of BUMPT cells with either 50 or 100 μM concentration significantly attenuated Cisplatin-induced elevation in IL-6 and p-NF- κB compared to BUMPT cells treated with Cisplatin alone. Treating control BUMPT cells with PCA did not significantly change the levels of IL-6 and p-NF-κB expression (Figure 4A–C).

### 2.5. Effect of PCA on Cisplatin-Induced Apoptosis in BUMPT Cells

**Figure 5:**
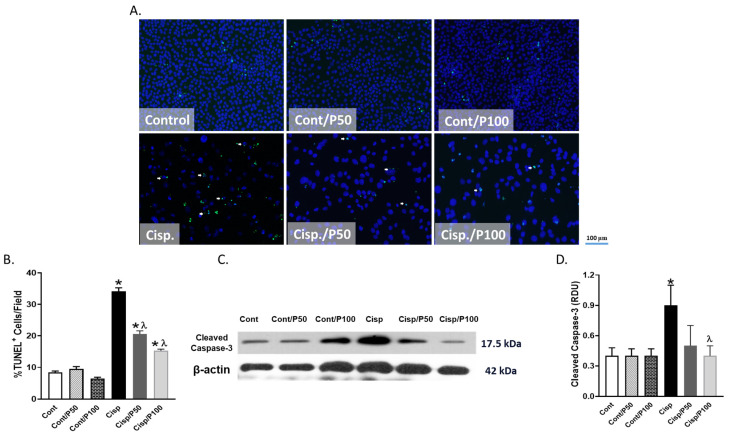
Effect of PCA on Cisplatin-induced changes in % of TUNEL^+^ cells (**A**,**B**) and cleaved caspase-3 expression (**C**,**D**). White arrows indicate TUNEL^+^ cells with nuclear counterstaining in blue (DAPI). Data represent means ± SEM (*n* = 7–8 per group). * indicates *p* < 0.05 vs. Cont, Cont/P50, or Cont/P100 and ^λ^ indicates *p* < 0.05 vs. Cisp.

Treating BUMPT cells with Cisplatin for 24 h significantly increased the % of TUNEL-positive cells and cleaved caspase-3 expression compared to the control group (Figure 5A–D). Although treating BUMPT cells with PCA at either 50 or 100 μM significantly reduced the elevation of TUNEL-positive cells in Cisplatin-treated cells, only PCA treatment at 100 μM did significantly decrease cleaved caspase-3 expression compared to BUMPT cells treated with Cisplatin alone (Figure 5A–D). Treating control BUMPT cells with PCA did not significantly affect the % of TUNEL-positive cells or cleaved caspase-3 expression (Figure 5A–D).

## 3. Discussion

Previous findings suggest that the cytotoxic effects of Cisplatin could be linked, at least in part, to ROS production [21]. Intracellularly, chloride atoms in Cisplatin are replaced with water molecules producing a highly electrophilic molecule that can bind thiol groups on cellular proteins and nitrogen on DNA [22]. Cisplatin can also accumulate in the mitochondria, leading to a further increase in ROS production [23]. Previous studies have shown a Cisplatin-induced increase in ROS in BUMPT cells, human proximal tubular (HK-2) cells, and various prostate and ovarian cancer cell lines [23,24,25,26]. In line with previous studies, our data suggest that Cisplatin induced ROS production in BUMPT cells after 24 h treatment. The antioxidant PCA decreased ROS production in a murine model of Cisplatin-induced nephrotoxicity [27] through the inhibition of NADPH-oxidase [27,28]. We have recently shown that PCA reduced oxidative stress in a murine model of unilateral ureteral obstruction [20] since PCA possesses ROS-scavenging properties due to its polyhydroxy structure. Consistently with previous findings, PCA reduced Cisplatin-induced elevation in ROS production and TBARS as a marker of oxidative stress in BUMPT cells in the current study.

Cisplatin-induced ROS production could trigger the activation of the cytoplasmic NF-κB inflammatory signal by phosphorylation and its translocation to the nucleus, which could further increase the expression of pro-inflammatory cytokines such as IL-6 [11]. In a previous study, Cisplatin was shown to increase the activation of NF-κB in BUMPT cells, which triggered the activation of the inflammatory cytokines IL-1β and TNF-α [26]. In the current study, Cisplatin also increased p-NF-κB and IL-6 levels in BUMPT cells. PCA has previously been proven to decrease the expression of NF-κB in non-small-cell lung cancer (NSCLC) cell lines, A549, H3255, and Calu-6 cell lines, and the decreased NF-κB expression was associated with a reduction in IL-6 [29]. Furthermore, PCA also decreased NF-κB in LPS-treated mouse BV2 microglial cell lines and LPS-treated HaCaT cells and primary keratinocytes [30,31]. In the current study, PCA decreased Cisplatin-induced elevation in p-NF-κB and IL-6 in BUMPT cells. It remains unclear if PCA could directly inhibit NF-κB phosphorylation and the subsequent activation of IL-6 in Cisplatin-treated BUMPT cells or if this effect of PCA is indirect via decreasing ROS production, which could then decrease NF-κB activation by phosphorylation and the subsequent IL-6 production.

Cisplatin-induced apoptosis was previously reported in vivo in murine kidneys [32] and testes [5] and in vitro in BUMPT cells [33,34], where the elevation in ROS induced by Cisplatin treatment could be the driving force for the increases in both DNA and mitochondrial damages. Treating BUMPT cells with Cisplatin also increased apoptosis in the current study as evidenced by the increased executioner expression of cleaved caspase-3 and the % of TUNEL^+^ cells. PCA was shown to provide anti-apoptotic activity in murine models of myocardial ischemia–reperfusion [35], methotrexate-induced epididymal and testicular toxicity [36], unilateral ureteral obstruction, and cardiac apoptosis [37]. Similarly, we have recently shown that PCA treatment reduced renal apoptosis in both sexes of a mouse model of unilateral ureteral obstruction [20].

Tight junctions are specialized intercellular junctions that play a crucial role in maintaining the integrity and barrier function of epithelial and endothelial cell layers in tissues [38]. These junctions create a seal between adjacent cells, limiting the passage of ions, molecules, and even cells through the intercellular space [39]. Tight junctions are particularly important in tissues where a barrier is needed, such as in the epithelial lining of the digestive tract, urinary tract, and blood vessels [40]. In proximal tubules, tight junctions are crucial for maintaining the barrier function and selective permeability of proximal tubular cells, facilitating the reabsorption of water and solutes while preventing nutrient leakage [41]. It is worth noting that this is the first study to investigate the physical barrier and the effects of Cisplatin on barrier integrity in BUMPT cells.

ZO-1 is a protein that plays a crucial role in the structure and function of tight junctions in epithelial cells. ZO-1 is a key scaffolding protein that contributes to the assembly and maintenance of tight junctions as it interacts with other proteins, such as occludins and claudins, to form a complex network that constitutes the tight junction strands [42]. Thus, ZO-1, through its association with other tight junction proteins, helps in creating a physical barrier between adjacent cells which will regulate the para-cellular transport of ions, water, and solutes and prevent the free passage of substances between cells [43].

Cisplatin treatment has been associated with the disruption of tight junctions in various cell types, including epithelial cells [44]. This disruption may result in the increased permeability of the epithelial barrier and could decrease the expression levels of tight junction proteins such as ZO-1 [45]. Cisplatin has previously been shown to decrease the expression levels of ZO-1 in a murine model of nephrotoxicity [45]. Since the elevation in oxidative stress can disrupt the function of tight junction proteins [46], the decreased ZO-1 expression in Cisplatin-treated BUMPT cells could be a consequence of elevation in oxidative damage induced by Cisplatin [47,48,49,50,51,52]. In line with previous studies, Cisplatin decreased the expression of ZO-1 in BUMPT cells in the current study, which then decreased the physical barrier as seen by decreased normalized resistance.

Studies suggest that PCA could restore barrier function. For example, PCA increased the expression of ZO-1 in the intestines of LPS-induced piglets [43], and in a murine model of dextran sulfate-induced ulcerative colitis [51]. PCA increased ZO-1 expression and improved the blood–brain barrier after intracerebral hemorrhage and in bEnd.3 cells [52]. In line with these studies, PCA also increased expression in ZO-1 and improved the physical barrier in BUMPT cells treated with Cisplatin in the current study. It is worth mentioning that the activation of NF-κB has been shown to be linked to the decreased expression of ZO-1 in bEnd.3 cells [53].

We could postulate that the elevation of oxidative stress triggers NF-κB inflammatory signal activation and increases apoptosis in BUMPT cells treated with Cisplatin, and these changes would functionally disrupt tight junction protein expression and barrier function. Thus, the interplay between oxidative stress, NF-κB activation, and elevation in apoptosis could be the driving force for the Cisplatin-induced disruption of barrier function in BUMPT cells, and the ability of PCA to reduce these changes could contribute to the regulation of tight junction integrity.

The current study provides evidence for the protective effects of PCA in Cisplatin-induced oxidative stress, inflammation, and apoptosis in BUMPT cells. Treating BUMPT cells with Cisplatin increased oxidative stress (ROS and TBARS levels), inflammation (IL-6 and p-NF-κB levels), and apoptosis (% of TUNEL^+^ cells and cleaved caspase-3 expression) relative to control cells, and these changes were associated with impairment in the tubular cell physical barrier function and a decrease in the tight junction protein ZO-1 expression. Treating BUMPT cells with PCA significantly reduced the Cisplatin-induced increases in oxidative stress, inflammation, and apoptosis in BUMPT cells, together with improvement in tubular cell physical barrier function and the expression of the tight junction protein ZO-1.

The findings of this study hold potential clinical implications. Given the widespread use of Cisplatin in cancer therapy and its dose-limiting nephrotoxicity, the identification of safe, effective nephroprotective agents is of great interest. PCA, a naturally occurring phenolic compound found in various fruits and vegetables, has demonstrated significant antioxidant and anti-inflammatory properties in both in vitro and in vivo studies. Our results suggest that PCA mitigates Cisplatin-induced oxidative stress, inflammation, and apoptosis in proximal tubular cells, key pathways involved in the pathogenesis of acute kidney injury. Its affordability, dietary availability, and favorable safety support its potential as a therapeutic adjunct to reduce renal toxicity in patients receiving Cisplatin, warranting further investigation in preclinical and clinical models.

## 4. Materials and Methods

### 4.1. Cells and Experimental Groups

BUMPT cells, which were originally established by Sinha, Wang [54], were kindly provided by Dr. Zheng Dong at Augusta University. BUMPT cells were cultured in DMEM with 10% FBS and 1% penicillin/streptomycin at 37 °C, 5% CO_2_, and used at passage 3 for all experiments to ensure consistency. Confluent BUMPT cells (70–80%) were treated with 20 μM Cisplatin ± PCA with different concentrations. The following groups were studied; **(1) Control (Cont)**: Normal cultured BUMPT cells; **(2) Cont/P50:** BUMPT cells treated with 50 μM PCA for 24 h; **(3) Cont/P100:** BUMPT cells treated with 100 μM PCA for 24 h; **(4) Cisplatin (Cisp)**: BUMPT cells treated with 20 μM Cisplatin for 24 h; **(5) Cisp/P50**: BUMPT cells co-treated with 20 μM Cisplatin plus 50 μM PCA for 24 h; and **(6) Cisp/P100:** BUMPT cells co-treated with 20 μM Cisplatin plus 100 μM PCA for 24 h. The concentration of Cisplatin was selected based on previous research showing its ability to induce significant apoptosis in BUMPT cells vs. control [34,55].

At the end of the experimental period, cell lysates were collected using ice-cold RIPA buffer from each dish. The cells were scrapped from the dishes and were further centrifuged to collect the supernatant. The collected supernatants were used for Western blotting and biochemical assessments. Each experiment was repeated at least 3 times.

### 4.2. 3-[4,5-Dimethylthiazol-2-yl]-2,5 Diphenyl Tetrazolium Bromide (MTT) Assay

An MTT assay was performed using the kit from Cayman (Cat. No. 10009365) according to the manufacturer’s instructions.

### 4.3. Quantification of Barrier Function

The barrier function was calculated in terms of normalized resistance using the electrical cell impedance system (ECIS) machine as previously published [56]. Briefly, 10 idf-96-well arrays (Applied Biosciences, Waltham, MA, USA) were first cleaned and modified using 50 µL/well of 10 mM L-cysteine for 15 min at room temperature. The wells were then washed twice with ultra-pure water before adding the attachment factor (50 µL/well). The arrays were washed once before 200 µL/well complete culture medium was added. After the wells’ stabilization, the arrays were removed from the holder and seeded with single-cell suspension in 200 µL/well complete cell culture medium (4 × 10^4^/well). After reaching steady state impedance, treatment started with Cisplatin ± PCA.

### 4.4. Western Blotting Assessments of Caspase-3 and ZO-1 Expression

Protein from cell lysates was quantified using the Bradford assay (Cat. No. 5000201), and 20 μg per sample was loaded onto 15-well gels (Bio-Rad, Cat. No. 4561096, Benicia, CA, USA). The proteins were separated via SDS-PAGE and transferred to PVDF membranes in 10% methanol transfer buffer. The membranes were blocked with 5% milk in PBST and incubated overnight at 4 °C with primary antibodies against caspase-3 (Invitrogen, MA1-16843, Waltham, MA, USA) and ZO-1 (Abcam, AB276131, Cambridge, UK). After PBST washes, HRP-conjugated secondary antibodies (Jackson ImmunoResearch, West Grove, PA, USA) and ECL substrate (Amersham) were used for detection. The membranes were stripped and reprobed for β-actin, and bands were quantified by densitometry using ImageJ (version 1.54; National Institutes of Health, Bethesda, MD, USA).

### 4.5. Immunocytochemistry

BUMPT cells were cultured on coverslips in 6-well plates (n = 3/group), fixed with 4% paraformaldehyde, and permeabilized with 1% Triton X-100. The cells were incubated overnight at 4 °C with IL-6 primary antibody (AF-406, Bio-Techne, Minneapolis MN, USA), followed by a fluorescent secondary antibody. IL-6 fluorescence intensity (mean/mm^2^) was quantified using ImageJ from images captured at 200× magnification (Carl Zeiss Axio Imager 2, Oberkochen, Germany).

### 4.6. Quantification of Thio-Barbituric Acid Reactive Species (TBARS) 

TBARS levels were quantified in the media of cultured BUMPT cells at the end of the experimental period as a marker of oxidative stress using a commercial kit from Cayman Chemicals (Cat. No. 10009055).

### 4.7. Reactive Oxygen Species (ROS) Assessments

ROS in BUMPT cells were assessed using 2,7 dihydrofluorscein diacetate staining (Cat. No. 20656, Sigma, St. Louis, MO, USA) as before [57]. Images were captured using Carl Zeiss Axio Imager 2 at 50× magnification. Relative fluorescence intensity was quantified using ImageJ analysis (version 1.54; National Institutes of Health, Bethesda, MD, USA).

### 4.8. Quantification of Phosphorylated Nuclear Factor Kappa Beta (p-NF-κB)

Phospho-NF-κB levels in BUMPT cells were quantified using a commercial ELISA kit from Rays-Bio (Cat. No. PEL-NFKBP65-S536) according to the manufacturer’s instructions.

### 4.9. TUNEL Assay

TUNEL staining was performed on BUMPT cells using the One-step TUNEL In Situ Apoptosis Kit (Elabsciences, FITC, Houston, TX, USA) per the manufacturer’s protocol. Images were captured at 100× magnification (Carl Zeiss Axio Imager 2). Green fluorescence indicated DNA damage/apoptosis, while DAPI marked nuclei. TUNEL-positive cells were quantified as a percentage of total cells per field using ImageJ.

### 4.10. Statistical Analysis

Data are expressed as means ± SEM. One-way analysis of variance (ANOVA) was used, followed by Tukey’s multiple comparison test as a post hoc test, and *p* <0.05 was considered statistically significant. Analyses were performed using Graph-Pad Prism version 9.0 software (Graph-Pad Software Inc., La Jolla, CA, USA).

## Data Availability

The datasets generated during and/or analyzed during the current study are available from the corresponding author upon reasonable request.
